# African Americans and European Americans exhibit distinct gene expression patterns across tissues and tumors associated with immunologic functions and environmental exposures

**DOI:** 10.1038/s41598-021-89224-1

**Published:** 2021-05-10

**Authors:** Urminder Singh, Kyle M. Hernandez, Bruce J. Aronow, Eve Syrkin Wurtele

**Affiliations:** 1 Bioinformatics and Computational Biology Program, Iowa State University, Ames, IA 50011 USA; 2 Center for Metabolic Biology, Iowa State University, Ames, IA 50011 USA; 3 Genetics Development and Cell Biology, Iowa State University, Ames, IA 50011 USA; 4 Department of Medicine, University of Chicago, Chicago, IL 60637 USA; 5 Center for Translational Data Science, University of Chicago, Chicago, IL 60637 USA; 6 Biomedical Informatics, Cincinnati Children’s Hospital Medical Center, Cincinnati, OH 45229 USA

**Keywords:** Computational biology and bioinformatics, Cancer genomics, Gene expression, Genomics, Cytokines, Inflammation

## Abstract

The COVID-19 pandemic has affected African American populations disproportionately with respect to prevalence, and mortality. Expression profiles represent snapshots of combined genetic, socio-environmental (including socioeconomic and environmental factors), and physiological effects on the molecular phenotype. As such, they have potential to improve biological understanding of differences among populations, and provide therapeutic biomarkers and environmental mitigation strategies. Here, we undertook a large-scale assessment of patterns of gene expression between African Americans and European Americans, mining RNA-Seq data from 25 non-diseased and diseased (tumor) tissue-types. We observed the widespread enrichment of pathways implicated in COVID-19 and integral to inflammation and reactive oxygen stress. Chemokine CCL3L3 expression is up-regulated in African Americans. GSTM1, encoding a glutathione S-transferase that metabolizes reactive oxygen species and xenobiotics, is upregulated. The little-studied F8A2 gene is up to 40-fold more highly expressed in African Americans; F8A2 encodes HAP40 protein, which mediates endosome movement, potentially altering the cellular response to SARS-CoV-2. African American expression signatures, superimposed on single cell-RNA reference data, reveal increased number or activity of esophageal glandular cells and lung ACE2-positive basal keratinocytes. Our findings establish *basal prognostic signatures* that can be used to refine approaches to minimize risk of severe infection and improve precision treatment of COVID-19 for African Americans. To enable dissection of *causes* of divergent molecular phenotypes, we advocate routine inclusion of metadata on genomic and socio-environmental factors for human RNA-sequencing studies.

## Introduction

The COVID-19 pandemic has infected over 31 million people and killed over 970,000 worldwide as of September, 2020 (https://coronavirus.jhu.edu/map.html). Its causative agent, the novel SARS-CoV-2, is an enveloped single stranded RNA virus that infects tissues including epithelial cells in the upper respiratory tract, lung alveoli, GI tract, vasculature endothelium, renal tubules, central nervous system, and myocardium^1–6^. The complex combinations and severities of symptoms caused by SARS-CoV-2 include fever, cough, fatigue, dyspnea, diarrhea, thrombosis, stroke, acute respiratory failure, renal failure, cardiac failure; in some individuals these may lead to long-term disability or death^2,5,6^. Differing patterns of disease may result from direct cellular infection, secondary inflammatory repercussions, and circulating immune and necrotic complexes from distal sites of infection and response^7–10^. Individuals who suffer the most severe sets of symptoms are more likely to be over 65 years of age, and/or have obesity or preexisting comorbidities such as diabetes, hypertension and heart disease^11^. How these attributes confer risk of increased disease severity to individuals is not well understood^4,8,10,12,13^. Identifying individuals most at-risk for severe COVID-19 infection, and determining the molecular and physiological basis for this risk, is critical to enable more informed public health decisions, and improving our identification and use of precision interventions.

COVID-19 cases and deaths are disproportionately higher among African Americans in the US relative to European Americans^12,14^. This disparity is caused in part by complex combinations of socio-economic factors, including underlying comorbidities, air quality, population density, and health care access^12^; heritable factors in the human host also influence COVID-19 symptoms^15–19^. To date, several genetic determinants of COVID-19 severity have been partially elucidated. Genetic variants of Angiotensin-Converting Enzyme2 (ACE2), a major human host receptor for the SARS-CoV-2 spike protein, may be linked to increased infection by COVID-19^18^. Human Leukocyte Antigen (HLA) gene alleles have been associated with susceptibility to diabetes and SARS-CoV-2^17^. A COVID-19 association at locus 9q34.2 spans several genes related to COVID-19, including blood type^16^. The genetic propensity in southern European populations for mutations in the pyrin-encoding Mediterranean Fever gene (MEFV) has been proposed to be associated with elevated levels of pro-inflammatory molecules, a cytokine storm, and greater severity of COVID-19^19^. Multiple GWAS associations based on ancestry are beginning to emerge (https://grasp.nhlbi.nih.gov/Covid19GWASResults.aspx)^16^.

Gene expression is a reflection of a cell’s composition and its spatial and developmental context in an organism. Modifying factors that determine gene expression span genetics, and physiological, environmental, and socio-environmental influences. In this study we seek to investigate potential differential expression of genes and pathways that may impact the severity of COVID-19 infection in African Americans. Research with macrophage cell lines has identified ancestry-related differences in innate immune response to bacterial pathogens, with cell lines isolated from individuals with African ancestry more likely to exhibit stronger inflammatory responses^20^. However, studies on the impact of Covid-19 mostly lack in sufficient numbers of individuals of different populations to achieve a high resolution analysis of differential expression responses.

Here, we utilize diverse, publicly-available datasets from 25 tissue-types to explore gene expression differences between African American and European American individuals. Specifically, we analyze -Seq data of “non-diseased” tissues from the Genotype Tissue Expression (GTEx, https://gtexportal.org/home/) project. And, as representative of highly perturbed systems, we analyze tumor samples from The Cancer Genome Atlas (TCGA, https://www.cancer.gov/about-nci/organization/ccg/research/structural-genomics/tcga); tumor tissue-types are particularly important because cancer confers an increased risk for severe outcomes from COVID-19^21^. Further, we seek to unravel the cellular origins of ancestry-associated differential gene expression through the use of Human Cell Atlas single-cell datasets from esophagus and lung tissues.

Taken together, our analyses reveal consistent differences between European Americans and African Americans in pathways, genes, and cell types likely to impact the severity of COVID-19. In esophagus and lung, two tissues critical to early SARS-CoV-2 infections, differential gene signatures between African-American and European American populations implicate specific cell lineages that are likely to alter viral disease severity. The results provide a critical baseline in the context of cellular and organismal health and resilience to disease from which to assess COVID-19 gene expression studies from a population perspective. Finally, we highlight the importance of evaluating population-related impacts on gene expression in the combined light of socio-environmental and genetic factors.

## Results

In order to identify genes differentially-expressed (DE) between African American and European Americans, we constructed an aggregated dataset of 7142 RNA-Seq samples encompassing non-diseased tissues from GTEx and tumors from TCGA^22,23^. The batch-corrected and processed data^23^ enable comparison across samples, and the large sample-size increases statistical power of the analysis. Race assignments are self-reported in the metadata; however, many of the individuals identifying as a single race may be from an admixed population^24,25^. We analyzed data and metadata using MetaOmGraph (MOG)^22^, software that supports interactive exploratory analysis of large data to identify and distinguish patterns across multiple dimensions (Table 1 and Supplementary Table S1).

### Multiple genes are DE between populations in a tissue- and tumor-specific manner

DE genes were identified for each tissue-type, as well as for pooled TCGA and GTEx data (Supplementary Tables S2-S28). To test for potential confounding factors that might explain gene expression pattern differences, we scrutinized differences between African American and European Americans populations controlling for biologically-relevant factors (sex, age, tissue-type, Body mass index (BMI) (as available in metadata), and cancer sub-type (as available in metadata)); under these analysis, DE genes from each Mann–Whitney (MW) analysis retained statistical significance in the corresponding limma model (Supplementary Tables S29-S55; Additional File 1). We used Hartigans’ dip test to each gene to evaluate bi- or multi-modality in gene expression distributions (Additional File 2). For a given gene and tissue-type, a bimodal structure could imply presence of underlying hidden variables that affect expression of that gene, such as unreported sub-population structure or environmental factors.

These analyses indicate there are numerous genes DE more than twofold between African American and European American populations (Table 1 and Supplementary Tables S2-S2). The analyses cannot distinguish as to whether these differences in expression are associated with socio-environmental factors or genetic factors, because this information is not included in the available metadata. Only tissue-types with over 15 African American individuals sampled showed DE genes > 2-fold difference in expression based on Mann–Whitney U test (BH-corrected *p* value $<0.05$).

**Table 1:** Number of DE genes in African Americans (AA) compared to European Americans (EA) in nine non-diseased tissue types and eight tumor types.

Project	tissue-type	#AA samples	#EA samples	#Upreg.	#Downreg.
GTEx	Breast	12	75	0	0
GTEx	Prostate	13	89	0	0
GTEx	Uterus	13	68	0	0
GTEx	Liver	15	97	0	0
GTEx	Stomach	29	159	4	6
GTEx	Colon	41	292	13	9
GTEx	Esophagus	80	564	19	11
GTEx	Thyroid	43	267	25	30
GTEx	Lung	39	269	45	20
TCGA	Lung squamous cell carcinoma (LUSC)	28	337	2	0
TCGA	Thyroid carcinoma (THCA)	25	292	3	3
TCGA	Lung adenocarcinoma (LUAD)	48	368	16	5
TCGA	Kidney renal papillary cell carcinoma (KIRP)	49	166	19	13
TCGA	Uterine Corpus Endometrial Carcinoma (UCEC)	54	70	28	5
TCGA	Colon adenocarcinoma (COAD)	54	188	30	21
TCGA	Kidney renal clear cell carcinoma (KIRC)	46	410	68	94
TCGA	Breast invasive carcinoma (BRCA)	142	674	83	164
GTEx	Pooled GTEx samples	292	1905	12	11
TCGA	Pooled TCGA samples	497	3238	13	21

Only tissue-types with AA sample size 12 or greater are shown. Samples are sorted first by project, and then by the number of upregulated genes. The number of samples affects the power of the DE test. Criteria for DE:>2-fold difference in expression based on Mann–Whitney U test (BH-corrected *p* value $<0.05$).

### Differences in gene expression between populations are enriched for the broad network of infection, inflammation, endosomal development, and ROS metabolism

GO terms related to the interrelated biological processes of inflammation/cytokines, endosomal development, and ROS metabolism are overrepresented among those genes that are DE between African Americans and European Americans (Supplementary Table S56).

**Figure 1:**
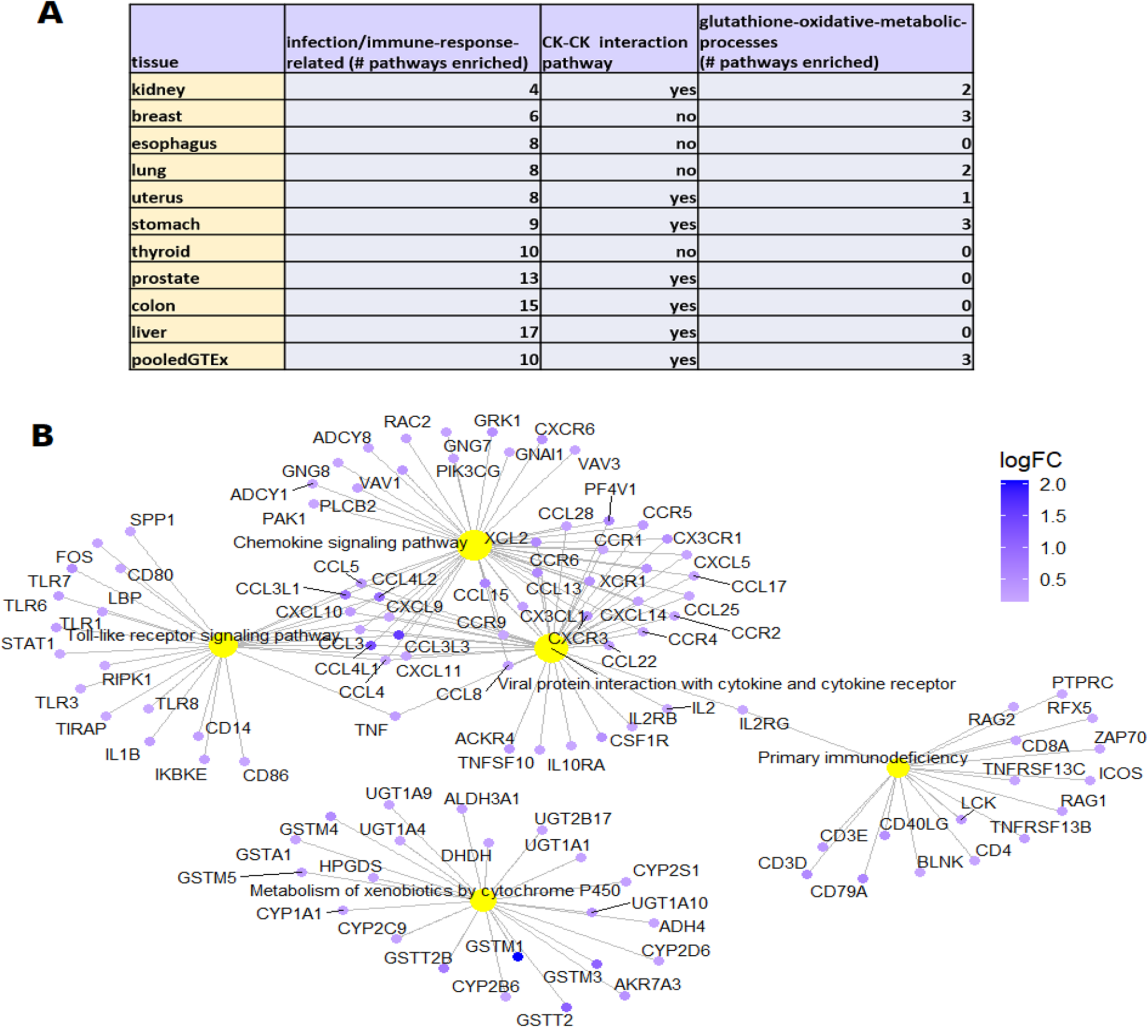
Gene Set Enrichment Analysis (GSEA) enrichment of KEGG pathways in African Americans compared to European Americans in pooled GTEx data. GSEA comprehensively analyses data for expression of all genes, rather than only the DE genes. (**A**) The most common pathways enriched among upregulated genes in African Americans for tissue-types in GTEx. See Additional File 3 for complete list of enriched pathways in 25 tissue-types. CK-CK, cytokine-cytokine receptor interaction; glutathione-oxidative metabolism includes (oxidative) metabolism of xenobiotics. The full enrichment analysis for each tissue-type is shown in Supplementary Table S57–S59. (**B**) The five most highly enriched pathways among upregulated genes of pooled samples from all tissue-types in GTEx are: Tol-like receptor signaling; chemokine signaling; primary immunodeficiency; viral protein interaction with cytokine and cytokine receptor; metabolism of xenobiotics by cytochrome P450.

Similarly, Gene Set Enrichment Analysis (GSEA) of all of the 25 GTEx and TCGA tissue-types shows KEGG pathways^26^ of immune- and inflammation-related processes are highly enriched (Supplementary Table S57–S58); the single most commonly-enriched pathway (found in 19 of the 25 tissue-types) is “cytokine-cytokine receptor interaction”; glutathione-oxidative processes of ROS and xenobiotic metabolism are enriched in nine tissue-types (Fig. 1A and Additional File 3). For example, analysis of pooled GTEx data detects coordinated changes between African Americans and European Americans associated with four cytokine-related pathways and oxidative drug metabolism (Fig. 1B and Supplementary Table S57).

Multiple genes are DE between African American and European American populations (Supplementary Table S2–S25). However, seven genes are highly and consistently DE between African Americans vs European Americans across all or most tissue-types. These are: C-C Motif Chemokine Ligand, CCL3L3; mitochondrial Glutathione-S-Transferase, GSTM1; Nuclear Pore Complex Interacting Protein Family Member, NPIPB15; Coagulation Factor VIII Associated genes, F8A3 and F8A2; FAM21B; and serine protease, PRSS21. Of these, four, C-C Motif Chemokine Ligand, CCL3L3; mitochondrial Glutathione-S-Transferase, GSTM1, F8A3 and F8A2, are directly related to the interrelated processes of infection, inflammation, endosomal motility, and ROS metabolism.

#### Cytokines, ROS and the storm

**Figure 2:**
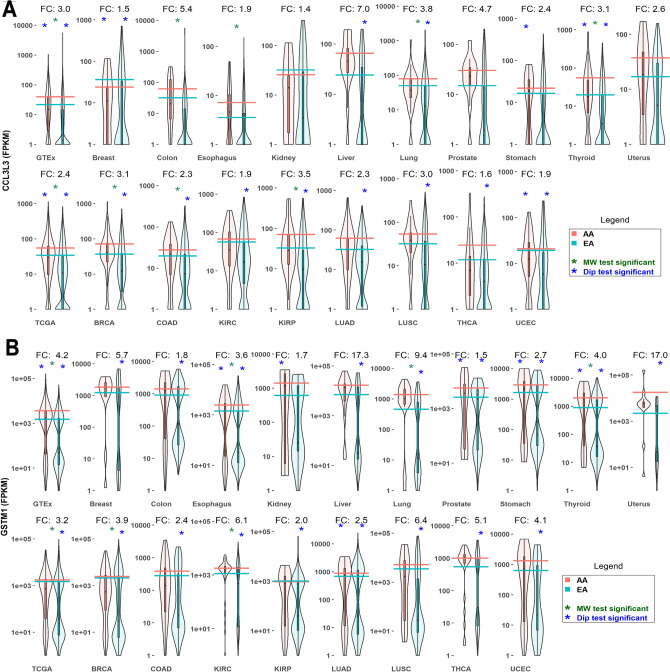
Upregulated expression of chemokine **CCL3L3** and mitochondrial glutathione-S-transferase **GSTM1** in African Americans compared to European Americans across multiple conditions. (**A**) **CCL3L3** is more highly expressed in African Americans over a wide range of tissue-types. CL3L3 binds to chemokine receptor proteins CCR1, CCR3, and CCR5. (**B**) **GSTM1** is more highly expressed in African Americans over a wide range of tissue-types. GSTM1 is a key player in metabolism of ROS and xenobiotics. (See Supplementary Tables S2–S28 for complete DE analysis). Violin plots summarize expression over each sample across the two populations. *AA* African American, *EA* European American. Horizontal lines represent mean log expression. Green asterisk, Mann–Whitney (MW) test for DE significant (Benjamini–Hochberg (BH) corrected *p* value $<0.05$). Blue asterisk, Hartigans’ dip test. Expression distribution is influenced by differences in population sizes (significant *p* value $<0.05$). FC, fold change AA/EA. GTEx and TCGA violin plots represent the pooled samples from each project. DE were computed within MetaOmGraph (MOG)^22^, in MOG’s statistical analysis module; R scripts were executed interactively via MOG to generate the violin plots.

Among the DE cytokines, the small inducible chemokine, CCL3L3, is more highly expressed in African Americans by up to sevenfold in most diseased and non-diseased tissue-types (Fig. 2) (Supplementary Table S2–S25). Genes involved in common biological processes that are DE $<1.3$-fold change in one or more tissue-types include: CCL4L1, CCL4L2, CCL3L1, CXCL9, CXCL13, CXCL17, CXCL10, GRK1, VAV3, CCL21, CCL8, and CCL15 (Supplementary Table S2–S25).

Several genes that mitigate oxidative stress, an inducer of cytokines, are DE between African American and European American populations. In particular, GSTM1, a key enzyme of oxidative stress, is more highly expressed in African Americans than European Americans across multiple tissue-types, including over ninefold higher expression in lung (Fig. 2). Functionally-related genes that are DE $<1.3$-fold change in expression based on Mann–Whitney U test in one or more tissue-types include: GSTM3, GTTT1, GSTT2, GSTT2B, GSTM4, FMO2, GSTM5, and CYP2A46; the CCL3L3 chemokine receptor proteins CCR1, CCR3, and CCR5 are not significantly DE (Supplementary Table S2–S25).

#### F8As and endosome motility

Endosomal function and autophagy are implicated in COVID-19 and intimately intertwined with cytokine and ROS signaling^27–29^. One little-studied player implicated in early endosome motility^30^ is the F8A/HAP40 (HAP40) protein, encoded by three genes (F8A1, F8A2, and F8A3) in humans^31^. The three F8A proteins are identical in sequence, and thus likely have the same molecular function.

**Figure 3:**
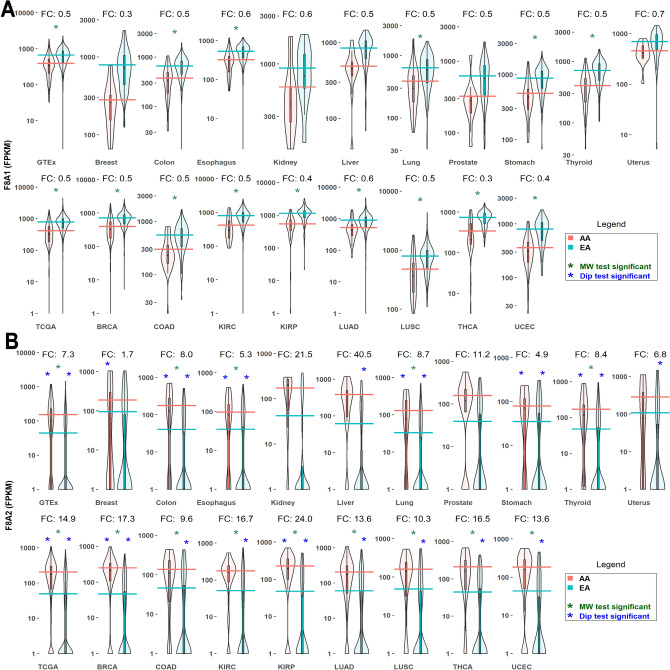
Differential expression of the HAP40 genes **F8A1** and **F8A2** in African Americans and European Americans across multiple tissue-types. HAP40 is a key molecular component of Huntington’s Disease, and shifts endosomal trafficking from the microtubules to actin fibers^30^. (**A**) **F8A1** expression is upregulated in European Americans. (**B**) **F8A2** expression is upregulated in African Americans. Violin plots summarize expression over each sample across the two populations. *AA* African American, *EA* European American. Horizontal lines represent mean log expression. Green asterisk, MW test for DE significant (BH corrected *p* value $<0.05$). Blue asterisk, Hartigans’ dip test. Expression distribution is influenced by differences in population sizes (significant *p* value $<0.05$). FC, fold change AA/EA. GTEx and TCGA violin plots represent the pooled samples from each project. DE were computed within MetaOmGraph (MOG)^22^, in MOG’s statistical analysis module; R scripts were executed interactively via MOG to generate the violin plots. (See Supplementary Fig. 2 for line plot comparison across individuals).

F8A1 is more highly expressed by about twofold in European Americans in almost every tissue-type analyzed (Fig. 3). Conversely, F8A2 and F8A3 are more highly expressed in African Americans. Expression of F8A2 in African Americans is up to 40-fold greater; expression of F8A3 is up to 6.6-fold greater. In LUSC, F8A2 and F8A3 are the only genes DE $>2$-fold (Supplementary Table S7). F8A2 and F8A3 follow a similar trend, being more highly expressed in African Americans (Fig. 3, Supplementary Fig. 1, and Supplementary Table S2–S25).

Distribution of F8A2 and F8A3 expression is bimodal in European Americans for most cancers, and part of the difference in levels of F8A2 and F8A3 expression between the two populations is due to their extremely low/undetectable levels of expression in a large proportion of the European American population.

Because of the vast differences in expression levels of the three HAP40-encoding genes between African Americans and European Americans, the paucity of literature on HAP40^32^, and the unclear relationships among F8A1, F8A2, and F8A3 genes, we further investigated the sequences, sequence variants, and the expression patterns of these genes.

The sequences of the HAP40-encoding proteins from F8A1, F8A2, and F8A3 are identical to each other in human reference genome GRCh38.p13 (https://www.ncbi.nlm.nih.gov/assembly/GCF_000001405.39). We searched for potential allele variants of HAP40 proteins encoded by F8A1, F8A2, and F8A3 in The Genome Aggregation Database (gnomAD)^33^. gnomAD assigns individuals to populations, by clustering of genetic features. Our search identified only very rare sequence variants in the HAP40s encoded by F8A1, F8A2, or F8A3 (gnomAD v3). No structural variants were identified for HAP40 of F8A1 or F8A3; a duplication of 54 aa is, very rarely, present in F8A2 (gnomAD SVs v2.1).

To our knowledge, F8A1, F8A2 and F8A3 gene expression has never been compared. This may be because expression of F8A2 and F8A3 genes is relatively low in most European Americans, and European Americans are the predominant population studied. Furthermore, most RNA-Seq studies report expression of *only* F8A1 or F8A3 (and not F8A2), presumably aligning all reads to one or the other gene.

We analyzed coexpression of the three F8A genes relative to the other 18,212 genes represented in the full TCGA-GTEx dataset using two statistical measures: Pearson’s correlation and Mutual Information (MI)^34^. Although the three F8A genes are proximately located on the X chromosome, their expression patterns are *not* correlated. F8A2 and F8A3 have a Pearson’s correlation of ($r=0.40$), while both are negatively correlated with F8A1. Indeed, of all 18,212 genes represented in the data, the expression pattern of F8A1 is most *negatively* (anti-) correlated with that of F8A2 ($r=-0.45$) and F8A3, ($r=-0.24$) (Supplementary Table S60). MI analysis indicates that F8A2 and F8A3 genes are more closely associated with F8A1 than with any other gene, consistent with the negative Pearson correlation (Supplementary Table S60). Also of note, F8A1 expression is not correlated with the F8 (Coagulation Factor FVIII) gene, although it resides with intron 22 of this gene.

### Signatures of DE genes correspond to specific cell types in esophagus and lung

We sought to determine whether genes differentially expressed between African Americans and European Americans corresponded to distinct cell populations present in the whole-tissue GTEx samples. This would provide information on cell-type representation across the two populations. To do this, we evaluated single cell datasets from two tissues highly relevant for SARS-CoV-2 infection: esophagus and lung^35,36^.

**Figure 4:**
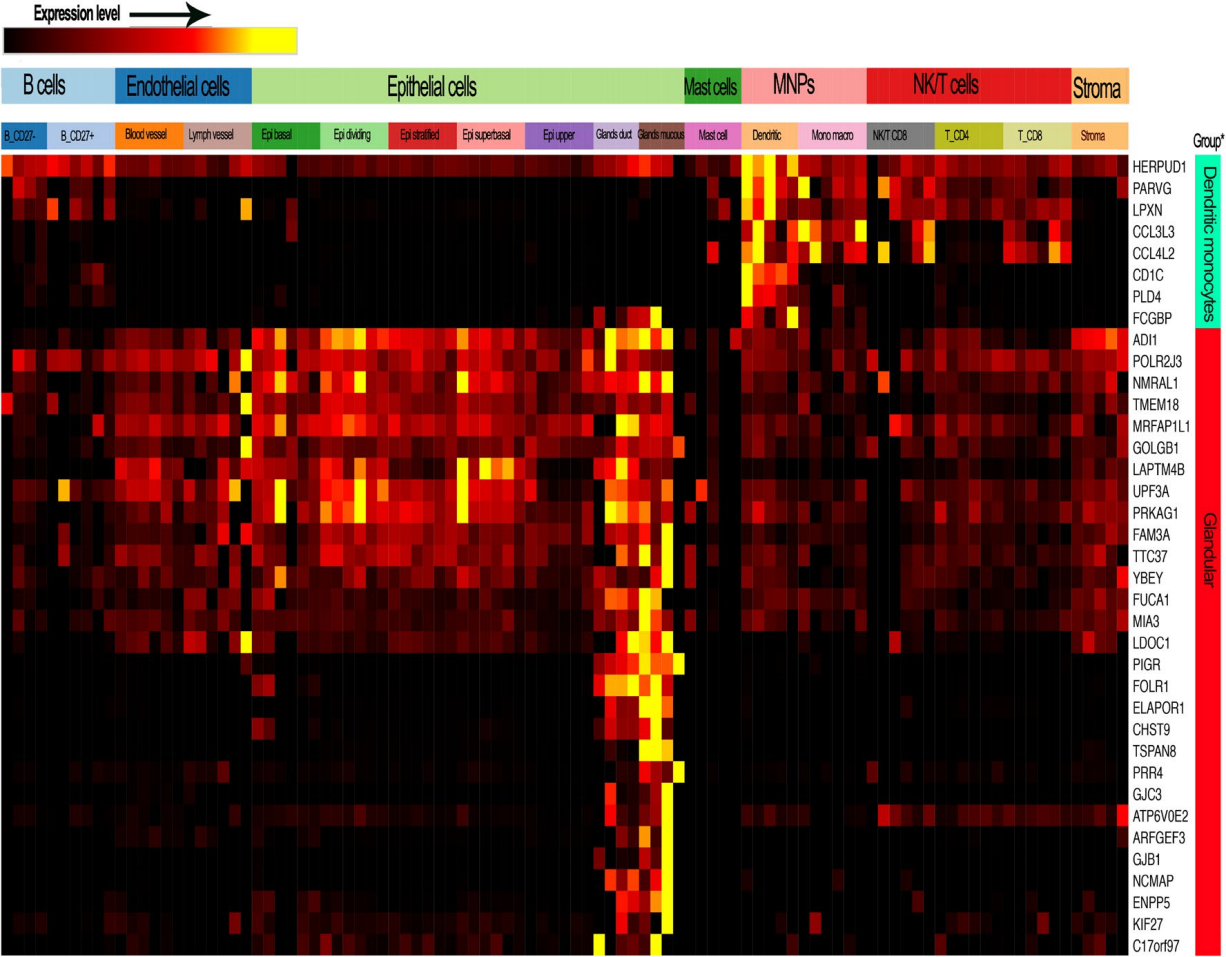
Esophageal genes that are differentially expressed in African Americans and European American samples correspond to genes known to be expressed in specific cell types. Genes upregulated in African American versus European American esophagus mapped to two cell lineages with prominent presence in the esophageal tissue stability dataset of the human cell atlas (https://data.humancellatlas.org/). One significant fraction of the African American-upregulated gene signature maps to glandular mucous epithelial cells of esophageal glands (genes marked by red, far right bar). Expression of several of the genes upregulated in African Americans is highly restricted to the mucous epithelial cells (TSAPN8, PRR4, ELAPOR1), whereas FOLR1, for example, is more highly expressed in the ductal epithelial cells of mucosal glands. A second, smaller, signature corresponds to hematolymphoid/myeloid lineage dendritic cells, as shown by CDC1C, PLD4, HERPUD1, and LPXN (genes marked by green, far right bar). In addition the genes that are most strongly expressed by those cell types, additional genes of the AA vs EA esophageal signature included several genes that are essentially exclusively expressed by those cell types. Toppcell-constructed gene modules (http://toppcell.cchmc.org) for each of the cell types reported to be present in the large scRNA-Seq dataset from esophagus^37^.

**Figure 5:**
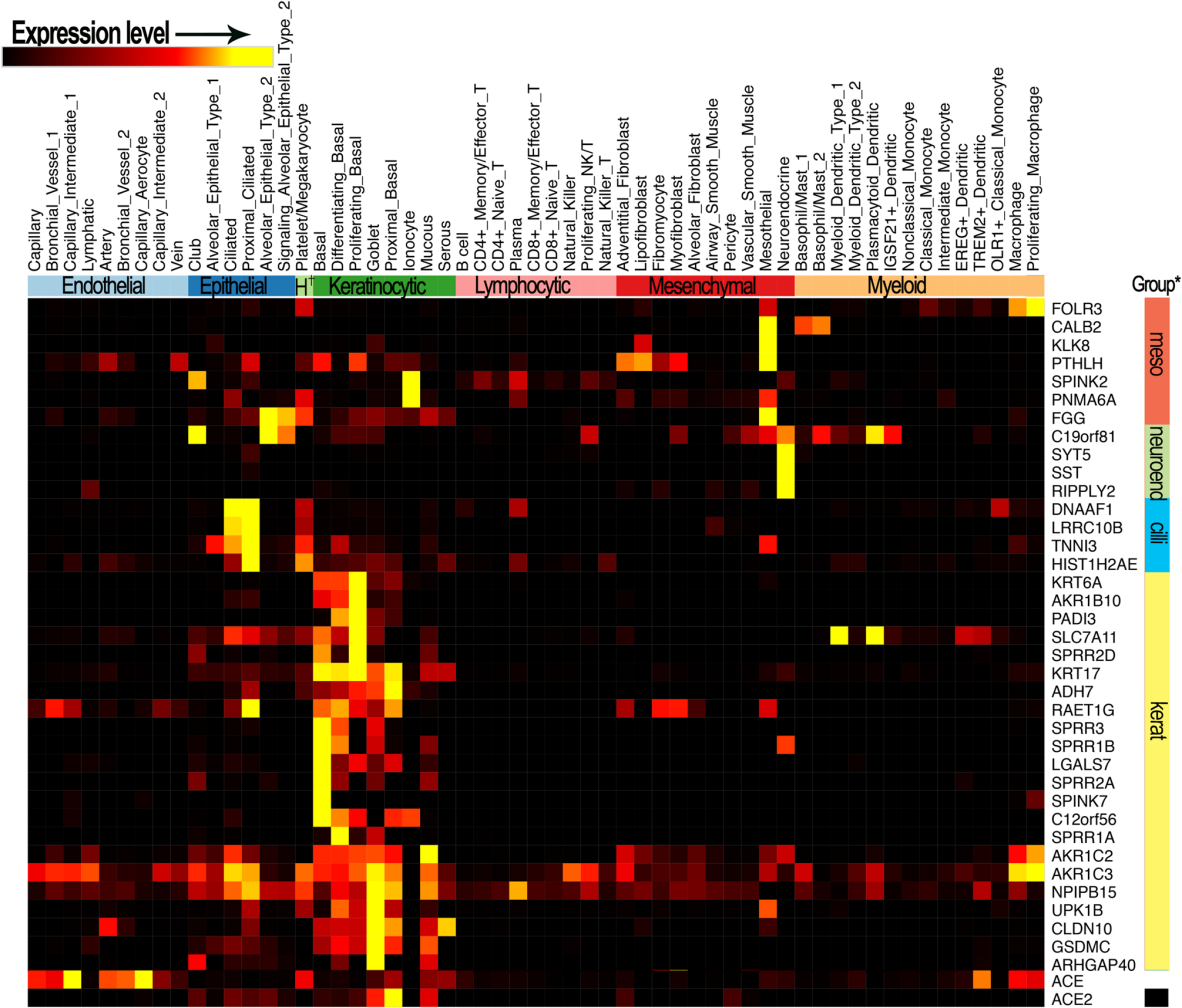
Lung gene signatures upregulated in African Americans versus European Americans map to proximal airway keratinocytic epithelial lineage, and to mesenchymal mesothelial and neuroendocrine cells. Marker genes for kerotinocytes (genes marked by yellow, far right bar); ciliated epithelial cells (genes marked by turquoise, far right bar); mesothelial mesenchymal cells (genes marked by red, far right bar); and neuroendocrine mesenchymal cells (mesenchymal). Note that the keratinocytic proximal basal epithelial cell is the cell subtype with the highest expression of ACE2 receptor, a major target of COVID-19 (ACE2 marked by black on bar at right). ToppCell-constructed gene modules (http://toppcell.cchmc.org) for each of the cell types reported to be present in the large scRNA-Seq dataset from lung^36^.

Genes upregulated in African Americans in the esophagus map predominantly to two cell lineages, glandular epithelial cells of esophagus glands, and hematolymphoid lineage-associated dendritic cells (Fig. 4). In proximal and distal airway cells of the lung, the signature of DE genes in African Americans versus European Americans corresponds to basal differentiating and proliferating keratinocytes (Fig. 5).

## Discussion

Human genetics contribute to the propensity and severity of diseases^25,38–45^. Sometimes the contribution is straightforward; a single allele variation found in Ashkenazi Jews, causes the vast majority of Tay-Sachs disease^44^. Sometimes it is more complex; for example, hypertension is more prevalent in African American than European American populations^45^ in part due to detrimental APOL1 mutations that are more frequent in West African populations^39^. Despite the paucity of studies focused on Western African populations, the propensity and severity of other diseases among this population have been attributed to genetics^25,39,46,47^.

In this study, we describe the molecular phenotypes, as revealed by differences in gene expression, in African Americans and European Americans across multiple non-diseased and diseased tissues. These distinct molecular phenotypes are likely caused by complex combinations of socio-environmental and genetic factors.

The predominant differences in gene expression, pathway enrichment, and cell-types between African Americans and European Americans are implicated in biological processes that highly impact COVID morbidity and mortality. These genes and pathways are not specific to COVID-19, but also would impact other diseases. Many COVID-19 deaths have been attributed to a cyclic over-excitement of the innate immune system^1,7,19^. This process, often termed a cytokine storm, results in a massive production of cytokines, and the body attacking itself rather than specifically destroying the pathogen-containing cells^1,7^. People with comorbidities, the elderly, and immunosuppressed individuals, may be at a greater risk for COVID-19 morbidity and mortality either because they may not respond to infection with a sufficient immune response^48^ and/or because they may be more likely to develop a cytokine storm^1,7^. Many cytokines and other immunomodulatory molecules are DE, and cytokine-related KEGG pathways are enriched, between African Americans and European Americans in one or more tissue-type.

The chemokine CCL3L3, upregulated in African Americans relative to European Americans under almost every diseased and non-diseased tissue-type we tested, and notably by 3.8-fold in lungs, is also upregulated in COVID-19-diseased human bronchoalveolar lavage fluid^49^. CCL3L3 encodes the CCL3 protein (also called MIP-1), a member of the functionally-diverse C-C motif chemokine family. A neutrophil chemotaxis protein, CLL3 acts as ligand for CCR1, CCR3, and CCR5, recruiting and activating neutrophils^10,50^. Neutrophils themselves are highly implicated in the severity of COVID-19^49,51,52^. CCL3 expression is upregulated in severe COVID-19^10,52^. Increase in accumulation of the CCL3 protein has been strongly associated with severe (but not mild) COVID-19 disease^53^.

GSTM1, more highly expressed in African Americans compared to European Americans in almost every tissue-type evaluated, is a key enzyme of mitochondrial ROS metabolism^54^. Mitochondrially-generated ROS induce expression of proinflammatory cytokines and chemokines, and are considered to play a key role in modulating innate immune responses against RNA viruses^54^ including SARS-COV-2^55^. GSTM1 itself is induced by nuclear factor erythroid 2-related factor 2 (Nrf2), a transcription factor that integrates cellular stress signals^56^. Increased expression of GSTM1, could lead to increased mitochondrial ROS, which might ultimately trigger inflammation and a cytokine storm^54^. Alternatively, increased GSTM1 expression might cause ROS to be metabolized rapidly, and prevented ROS from initiating a sufficient immune response. GSTM1 has a second critical function– in metabolism of xenobiotics, including many toxins and pharmaceuticals^54^. In the latter case, pharmaceuticals may be more rapidly metabolized and rendered inactive.

The most dramatic differences in gene expression in African Americans compared to European Americans are associated with the highly-conserved but little-studied F8A genes, which each encode the HAP40 protein. F8A1 is upregulated about twofold in European Americans. In contrast, F8A2 and F8A3 are even more highly upregulated in African Americans, and in over half of the samples from European American individuals, levels of expression of F8A2 and F8A3 were negligible.

Although coagulation factor VIII has a high frequency of mutations across populations^57^, we found the F8A1, F8A2 and F8A3 genes and CDSs to be highly conserved across populations (gnomAD v3). This conservation is consistent with the three genes having a similar and specific molecular function. However, despite their proximity and encoding the identical protein, F8A1, F8A2 and F8A3 each have highly distinct patterns of expression across the thousands of samples of tissues and cancers in the TCGA/GTEx dataset, indicating they may participate in different or overlapping biological scenarios.

HAP40 function has been researched mostly in the context of F8A1 and the critical role of that gene in slowing early endosome mobility in Huntington’s disease^31^. In Huntington’s, HAP40 forms a bridge between the huntingtin protein and the regulatory small guanosine triphosphatase, RAB5; formation of this complex reduces endosomal motility by shifting endosomal trafficking from the microtubule to the actin cytoskeleton^30^. F8A1 overexpression in striatal neuron cell lines from mice resulted in increased ROS and mitochondrial dysfunction^58^. Knockouts of F8A1 in human HeLa and HEK293 cells yield altered/reduced autophagy and shorter life spans^58^. Knockouts of the single F8A gene in Drosophila similarly show reduced activity, altered/reduced autophagy, and shorter lifespan^59^.

F8A1 expression is increased under several conditions, including Huntington’s disease^60^, presence of a SNP variant for type 1 diabetes risk^61^, cytotrophoblast-enriched placental tissues in women with severe preeclampsia^62^, and mesenchymal bone marrow cells as women age^63^. Its potential roles in the latter conditions has not been investigated.

Altered endosome motility would play an important but complex role in infection and the innate immune response, and might either promote or hinder the battle between SARS-CoV-2 and its human host^28,64^. Coronaviruses including SARS-CoV-2 mainly enter host cells via binding to the ACE2 receptor followed by endocytosis^7,65^. Nascent early endosomes are moved along the microtubule cytoskeleton, fusing with other vesicles; varied molecules can be incorporated into the endosomal membrane or its interior^28,64^. This regulated development enables diverse fates. For example, in the context of SARS-CoV-2, endosomes might release viral RNA or particles; they might merge with lysosomes and digest their viral cargo; or they might fuse with autophagosomes (autophagy) and subsequently with lysosomes that digest the cargo^28,64^. SARS-CoV-2 might reprogram cellular metabolism to suppress autophagy and promote viral replication^66^; conversely, the cell might modify autophagy machinery to decorate viral invaders with ubiquitin for eventual destruction, activate the immune system by displaying parts of the virus, or catabolize excess pro-cytokines. Autophagy might induce cytokine signaling, which could promote protective immune response or engender a destructive storm of cytokines, inflammation and tissue damage^28^. Because of its function in early endosome motility, HAP40 has implications as a potential molecular target in therapy of endosomal and autophagy-related disorders such as COVID-19.

Our results regarding differentially-expressed genes and biological processes are consistent with those of a study using cultured primary macrophages that had been isolated from individuals of African and European ancestry. This study identified thousands of genes with ancestry-associated differences in expression in response to bacterial infection, and additional evidence of underlying genetic control and population-specific signatures of adaptation^20^. Despite the disparity between the biological systems analyzed, the differentially expressed genes were similar (See Supplementary File 5).

Our study using single cell reference data indicate several cell type-specific associations of the signatures of DE genes in African Americans versus European Americans in esophagus and lung. This interrogation reveals enrichment of DE genes in immune-related cell-types. One model by which this might occur is that individuals of one population tend to have different proportions of a given cell type or histological structure. An alternative model is that individuals of one population might tend to maintain some of their cell types in a state of relatively higher activation. Either explanation would lead bulk RNA-Seq analyses, such as tissue-types from GTEX or TCGA, to demonstrate elevated expression of those transcripts in that population.

Although at a population level, major differences exist in expression of inflammation-related genes and cell-type-specific associations between African Americans and European Americans, when considered on the basis of each individual within each population, gene expression differences are more complex. Individuals within a population may exhibit all, no, or some portion of the prevailing differences in a population. That some genes show bimodal expression distribution in some tissue-types African American and/or European American populations further emphasizes this variation.

Thus, the significance of these patterns and their relationship to differential susceptibility or risk of severity from COVID-19 (or another disease) must be considered from nuanced perspectives. Importantly, it may be that only a fraction of the signature and a fraction of the individuals in a population are at elevated risk of more severe disease. In addition, different mechanisms of risk may be operative within different individuals within an population. For example, elevated abundance or activity of cells that are the target of COVID-19 (e.g., ACE2-positive basal keratinocytes) could lead to a greater infection burst during initial phases with a larger number of virions being released systemically. If, as it appears from the alignment of the DE genes in African Americans compared to European Americans to the lung single cell data, this is the case for African American-individuals, then they might be more readily taken over by infecting SARS-Cov2 virions.

The differential expression of genes implicated in COVID-19 morbidity and mortality between African Americans and European Americans reported herein emphasizes the importance of integrating gene expression data into the genetic and socio-environmental factors at a population level. Further, RNA-Seq data has been shown useful in clinical practice for pediatric cancers^67^, and this practice could be extended to other diseases. Our analysis, in concurrence with those of^20,68^, supports the concept that processes of disease and stress are enriched in comparisons of African American and European American populations, and this may be in part because ancestral selection pressures such as pathogens, temperature stress, and toxins, were very strong, and there were very different complements of these stresses in the regions where these two populations lived. To survive, humans living in Europe and those living in Western Africa would have had to evolve the ability to resist the diverse prevalent local pathogens and stresses. Other differences would be due to a difference in socio-environmental factors, such as stress, commorbidity, or exposure to pollution^69^.

Expression data provided a tremendous wealth of information from which researchers can model the factors that predict and determine disease. However, the utility of these data is reliant on adequate *representation of cohorts* and on sufficient *metadata* describing the individuals sampled. For example, ethnic bias, practical factors (such as subject availability), as well as a paucity of molecular medical research in many regions of the world often result in insufficient numbers of subjects from many populations being represented in medical studies^70,71^. This lack of representation greatly impedes the development of precision prognosis and therapy based on genetics^42,70^. For example, here, we were limited to comparison of differences between gene expression in African American and European American populations because even in the large GTEx and TCGA studies, sample sizes for the other three major population groups (Asian, Native American, and Pacific Islanders) were generally too low for robust statistical assessment (Supplementary Table S1).

In addition, even if sample sizes for race are sufficient, information on the ancestry of each individual sampled is needed. Self-reported metadata on race is often not publicly available for individual samples. However, methods of assigning ancestry to individuals sampled for RNA-Seq are being developed and applied^72,73^.

Finally, current pipelines for RNA-Seq analysis often represent only the more highly or consistently expressed annotated genes^74,75^. Population-specific genes may be missed in the analysis unless they are in the predominant population being studied. The same is true for members of genes families that are preferentially-expressed in particular populations. An example brought out by our study is the F8A2 gene, which is DE-up in African Americans compared to European Americans; however, F8A2 is not even represented in the processed data of many RNA-Seq studies.

Combined information on socio-environmental factors and genomics of individuals sampled is critical in dissecting the determinants of gene expression in that individual. Yet for humans, a dichotomy exists between socio-environmental and genomic investigations. Among the vast body of human RNA-Seq data deposited, not only are metadata on the ancestry of the sampled individuals often unavailable, but socio-environmental metadata are almost never present. Thus, apart from the pioneering sociogenomics research of^69,69,76^ and studies such as^68,77,78^, socio-environmental information are rarely considered in ’omics analyses. Indeed, because of the scant metadata on socio-environmental determinants it is not even possible to determine possible skewness of representation of socio-environmental groups among the individuals sampled; thus, socio-environmental factors represent high-impact complex hidden covariates that would be challenging to model.

Conversely, sociological studies rarely incorporate ’omics information. For example, the U.S.-based Robert Wood Johnson Foundation (https://www.rwjf.org/en/library/interactives/whereyouliveaffectshowlongyoulive.html) cites research that “your zip code can be more important than your genetic code” for your health; however, the analyses were done without actually evaluating genetic codes. Because socio-environmental data was absent in these studies, they were unable to distinguish genetic effects from socio-environmental causes.

In the current study, because of the lack of socio-environmental metadata, we are limited to reporting population-based differences (rather than ancestry-based differences or socio-environmental associations) in gene expression. The very real health benefits that can be gained from metadata access need to be more carefully balanced against privacy concerns. Without routine inclusion and availability of diverse metadata for human ’omics samples, data mining is hampered, and important medical information is lost.

## Conclusion

We have found that genes whose expression differs between African American and European American populations across multiple biological sample types and tissues are deeply associated with multiple pathways and cell types associated with infection, inflammation, environmental exposures, and immunologic and mucosal cell types that are central to targets-of and defenses-against COVID-19. These differences are evident despite the fact that race is self-reported in the metadata, and many Americans are racially admixed^25^. By highlighting the wide-ranging differences in expression of genes implicated in the morbidity and mortality of COVID-19 across populations, and by revealing apparent cell-type differences between populations, we provide baseline signatures that could factor genomics, environmental, and immunologic parameters to improve preventives and therapeutics essential to fight diseases such as COVID-19.

## Methods

### Datasets

We selected bulk RNA-Seq data for this study from Genotype Tissue Expression (GTEx, https://gtexportal.org/home/) and The Cancer Genome Atlas (TCGA, https://www.cancer.gov/about-nci/organization/ccg/research/structural-genomics/tcga). GTEx provides data representing “non-diseased” samples from diverse tissues. Non-diseased refers to the tissue itself, however, in some cases the individual sampled was postmortem and the causes of death are varied. TCGA project is the largest project available on different diseased samples (tumors) of multiple tissue origins. Both projects have metadata on the (self-reported) races of the individuals who contributed samples. These two projects provide a unique opportunity to evaluate differences in gene expression across populations in multiple tissue-types that vary by cite of collection and disease status. Tissue-types were selected for downstream analysis based largely on having sufficient numbers of individuals from each ancestry. (Even between African American and European American populations, not every “non-diseased” tissue or cancer tissue had sufficient samplings of African Americans for robust statistical assessment (Supplementary Table S1)). We refer to those self-reporting as “Black or African American” as “African Americans” and “White” as “European Americans”.

The data files and the precompiled MOG project, *MOG_HumanCancerRNASeqProject*, were downloaded from http://metnetweb.gdcb.iastate.edu/MetNet_MetaOmGraph.htm^22^. This project uses batch-corrected and processed data to enable comparison across samples^23^. *MOG_HumanCancerRNASeqProject* contains expression values for 18,212 genes, 30 fields of metadata detailing each gene, across 7,142 samples representing 14 different cancer types and associated non-tumor tissues (TCGA and GTEX samples) integrated with 23 fields of metadata describing each study and sample^22^.

### Statistical and correlation analyses

The MOG tool was used to interactively explore, visualize and perform differential expression and correlation analysis of genes.

The Mann–Whitney (MW) test was used to identify DE genes between two groups; we chose this non-parametric analysis as it makes no assumptions about the data distribution. We define a gene as DE twofold or more between two groups if it meets each of the following criteria:

1. Estimated fold-change in expression of twofold or more ($\log$ fold change, $|logFC| \ge 1$), where *logFC* is calculated as in limma^79^.)
2. Mann–Whitney U test is significant between the two groups (Benjamini–Hochberg (BH) corrected *p* value $<0.05$)

Pearson correlation values and Mutual Information values were computed after data was $log_2$ transformed within MOG, in MOG’s statistical analysis module. R scripts were written to create the violin plots; these scripts were executed interactively via MOG.

### Covariate evaluation

To check for potential sampling differences between populations that might confound the analysis, we fit linear models using limma^79^ in R, to adjust for for biologically relevant, potential confounding factors of race, gender, tissue/tumor type, age, and as metadata was available, BMI, and cancer subtypes. (Supplementary Table S29–S55).

Because ratios of cancer subtypes may differ between races (as reported for breast cancer in African American women)^42,80^), we evaluated the RNA-Seq data from African Americans and European Americans in BRCA samples for potential confounding effects due to different ratios of four breast cancer subtypes: basal-like (BAS), human epidermal growth factor receptor-2 positive/estrogen receptor negative (Her2), luminal A (LumA), and luminal B (LumB) (subtype information was collected using TCGABiolinks^81^); all genes DE with >2-fold change in MW analysis retained statistical significance in limma analysis of BRCA data, although the fold-change levels varied (Supplementary Table S41). Similarly, we included BMI, where it was available in the metadata, in the limma analysis (Additional File 1).

To assess whether a given distribution shows bi- or multi-modality we applied the Hartigans’ dip (Dip) test, using the R package diptest (https://cran.r-project.org/package=diptest) (Additional File 2).

### Gene expression enrichment

Overrepresentation of biological processes and other functional analysis was assessed at https://toppgene.cchmc.org/. Geneset enrichment analyses (GSEA) were performed using the clusterProfiler library in R^82^.

### Cell-type analysis

African American-vs-European American gene signatures were compared to cell type and compartment-specific gene signatures using the newly developed cell type specific gene modules available in the ToppGene tool^83^. The corresponding gene lists in ToppGene were derived from large-scale gene expression signature mining in this case of human cell atlas reference datasets from human esophagus and lung^35,36^ hosted in ToppCell (http://toppcell.cchmc.org. Heat map visualization of genes differentially-expressed by African Americans versus European Americans in each cell type module in the selected tissues was done using Morpheus (https://software.broadinstitute.org/morpheus/) using ToppCell’s “super binned” gene expression for each cell type within each single cell dataset.

## Supplementary information

Supplementary material 1

Supplementary material 2

Supplementary material 3

Supplementary material 4

Supplementary material 5

Supplementary material 6

Supplementary material 7

Supplementary material 8

Supplementary material 9

Supplementary material 10

## Data Availability

We subscribe to an open data model (https://www.go-fair.org/fair-principles/). MOG is free and open source software published under the MIT License. MOG software, user guide, and the *MOG_HumanCancerRNASeqProject* project datasets and metadata described in this article are freely downloadable from http://metnetweb.gdcb.iastate.edu/MetNet_MetaOmGraph.htm. MOG’s source code is available at https://github.com/urmi-21/MetaOmGraph/. Detailed information and code on how to reproduce the results, along with Additional files, are available at https://github.com/urmi-21/COVID-DEA. Supplementary data are available at https://github.com/urmi-21/COVID-DEA.
