# Supplementary material for: African Americans and European Americans exhibit distinct gene expression patterns across tissues and tumors associated with immunologic functions and environmental exposures
Source: Sci Rep. 2021 May 10;11:9905. doi: 10.1038/s41598-021-89224-1 (PMC8110974; doi:10.1038/s41598-021-89224-1)
Supplement: Supplementary file 10 — Supplementary material 10 [file 41598_2021_89224_MOESM10_ESM.pdf]

Data:
C-C Motif Chemokine Ligand CCL3L3, mitochondrial Glutathione-S-Transferase GSTM1; Nuclear Pore ComplexInteracting Protein Family Member NPIPB15, Coagulation Factor VIII Associated genes F8A3 and F8A2; FAM21B; and serineprotease PRSS21

Nedelec et al., 2016 https://doi.org/10.1016/j.cell.2016.09.025 - (EA and AA; Conditions: Listeria, Salmonella, Non-Infected, response Listeria, response Salmonella) - https://immunpop.com

Population differences in expression (based on FDR 0.05):

- CCL3L3
  - Listeria (5.6e-4; AA > EA)
  - Salmonella (2.7e-3; AA > EA)
  - Non-infected (1.6e-2; AA> EA)
- GSTM1
  - Listeria (4.9e-3; AA > EA)
  - Salmonella (8.3e-3; AA > EA)
  - Non-infected (8.4e-4; AA > EA)
- NPIPB15
  - Listeria (0.0e0; AA > EA)
  - Salmonella (0.0e0; AA > EA)
  - Non-infected (0.0e0; AA > EA)
- F8A1
  - Listeria (0.0e0; AA < EA)
  - Salmonella (2.2e-3; AA < EA)
  - Non-infected (0.0e0; AA < EA)
- F8A3
  - Listeria (0.0e0; AA > EA)
  - Salmonella (0.0e0; AA > EA)
  - Non-infected (0.0e0; AA > EA)
- F8A2
  - Listeria (0.0e0; AA > EA)
  - Salmonella (0.0e0; AA > EA)
  - Non-infected (0.0e0; AA > EA)
- FAM21B
  - Listeria (1.7e-2; AA < EA)
  - Salmonella (4.1 e-2; AA < EA)
  - Non-infected (2.9e-2; AA < EA)
- PRSS21
  - Listeria (0.0e0; AA < EA)
  - Salmonella (5.1e-4; AA < EA)
  - Non-infected (1.6e-4; AA < EA)

Pop diffs in isoform usage (based on FDR)

- CCL3L3
    - Listeria (1.7e-3)
    - Non-infected (1.1e-3)
- GSTM1 – NA
- NPIPB15 – NA
- F8A1 - NA
- F8A3 – NA
- F8A2 – NA
- FAM21B – Not significant
- PRSS21 - NA
